# Chronic TLR Signaling Impairs the Long-Term Repopulating Potential of Hematopoietic Stem Cells of Wild Type but Not Id1 Deficient Mice

**DOI:** 10.1371/journal.pone.0055552

**Published:** 2013-02-01

**Authors:** Ying Zhao, Flora Ling, Hong-Cheng Wang, Xiao-Hong Sun

**Affiliations:** 1 Immunobiology and Cancer Research Program, Oklahoma Medical Research Foundation, Oklahoma City, Oklahoma, United States of America; 2 Department of Cell Biology, University of Oklahoma Health Sciences Center, Oklahoma City, Oklahoma, United States of America; Emory University, United States of America

## Abstract

Hematopoietic stem cells (HSCs) maintain life-long blood supply but are inevitably exposed to various inflammatory stimuli, which have been shown to be harmful for HSC integrity but the mediators of the deleterious effects have not been fully identified. Here, we show that daily injection of mice with 1 µg of LPS for 30 days triggers a storm of inflammatory cytokines. LPS injection also stimulated the transcription of the Id1 gene in HSCs in vivo but not in vitro, suggesting an indirect effect. To determine the effects of LPS treatment on HSC function and to evaluate the significance of Id1 expression, we assess the repopulating potential of wild type and Id1 deficient mice, which were subjected to a 30 day regimen of LPS treatment. We found that LPS caused dramatic reduction in the long-term but not short-term repopulating activity of wild type but not Id1 deficient HSC. This treatment also led to increases in HSC counts, decreases in BrdU-label retention and disturbance of quiescence detected by Ki67 staining in wild type but not Id1 deficient mice. Together, it appears that Id1, at least in part, plays a role in LPS-induced damage of HSC integrity.

## Introduction

Adult hematopoietic stem cells (HSCs) are responsible for replenishing all blood lineages throughout the lifespan of an individual. Well-orchestrated programs are in place to balance HSC differentiation and self-renewal to meet this constant, life-long demand [1]. Recent advances in flow cytometric analysis and separation, gene expression profiling and functional assays have provided better understanding of stem cell biology in normal situations. However, stem cells in living organisms are also subjected to various environmental insults from pathogens and inflammatory cytokines, which will undoubtedly impact the maintenance and function of HSCs. How stem cells respond to these insults and what molecular events control these responses are unanswered questions.

Long-term hematopoietic stem cells (LT-HSC) are rare populations of cells representing approximately 0.003% of the total bone marrow cells in the mouse [2]. Because of the paucity of these cells, their identification and purification have been extremely challenging. A widely used approach for isolating stem cells had been to obtain the lineage negative (Lin^−^) c-kit^+^Sca-1^+^ fraction (LSK) [3], [4]. However, only 1% of this population constitutes LT-HSC [5]. Recent advancements have provided a more accurate definition of LT-HSC, which can be described as Lin^−^c-kit^+^Sca-1^+^CD150^+^CD48^−^ [2]. LT-HSC can also be enriched by isolating CD34^−^Flt3^−^LSK [6]. However, the ability to repopulate irradiated recipient mice by various donor fractions of the bone marrow remains to be the gold standard for stem cell activity, as well as for the estimation of stem cell frequency [7]. Nevertheless, it is now possible to better evaluate stem cell properties by determining both the number and repopulating potential of stem cells in any given situations.

Two of the fundamental issues concerning HSC biology are the maintenance of their “stemness” and the ability to self-renew. Although stem cells have unique properties, fundamental cellular processes occurring in all cell types, such as proliferation, differentiation and survival are also key events controlling stem cell integrity. Therefore, their molecular regulation may be mediated by factors also utilized by other cell types. For example, like their roles in more differentiated cells, c-myc and N-myc are necessary for HSC proliferation during homeostasis [8], [9]. Another example is the cell cycle regulator, p21, which is known to be responsible for keeping somatic cells in a quiescent state [10], [11]. When p21 is deleted, HSCs hyper-proliferate under normal homeostatic conditions but become exhausted upon bone marrow injury [12].

Several members of the basic helix-loop-helix family of transcriptional regulators have been implicated in regulating stem cell maintenance [13]–[18]. E proteins, encoded by the E2A, HEB, and E2-2 genes, are transcriptional activators that play crucial roles in lymphoid differentiation and also activate the transcription of cell cycle regulators such as p21 [19]–[22]. Genetic ablation of one of the E proteins, E47, or the entire E2A gene resulted in a significant reduction in the number of short-term HSC or multipotent progenitors, suggesting a critical role for E proteins in the differentiation of HSCs. [17], [18] E2A deficiency also impaired long-term repopulating activity of stem cells in serial transplant assays [18], [23]. The function of E proteins can be hampered by inhibitory HLH proteins including Id (Id1–4), which diminish the DNA binding activities of E proteins [24]–[26]. We have previously shown that Id1 is expressed in LT-HSC, and Id1 but not Id3 deficiency leads to a reduction in the number of LT-HSC and the repopulating potential of bone marrow cells [13]. In contrast to E protein deficiency, short-term repopulating activity was not impaired. Id1 has also been shown to play an important role in supporting the microenvironment of HSC, which is consistent with the fact that Id1 along with Id2 and Id3 are expressed in osteoblasts and endothelial cells [27]–[29]. In addition, Tal1/SCL and Lyl1 are also bHLH proteins capable of associating with E proteins and function as transcription activators or inhibitors depending on the context. These proteins play redundant roles in supporting HSC survival [30]–[32].

Inflammation represents an innate cellular response to a variety of harmful stimuli and pathological conditions [33]. Hematopoietic stem cells residing in the bone marrow or circulating in the blood stream or tissues have opportunities to directly encounter bacteria or virus and to be exposed to inflammatory cytokines secreted as a result of TLR stimulation [34]. Other inflammatory conditions such as autoimmunity can also affect hematopoiesis through a variety of cytokines. Therefore, despite these different circumstances, similar mechanisms may underlie the deleterious effects on HSC.

To examine the effect of chronic TLR signaling on HSC maintenance, Esplin et al. has developed a protocol to treat mice with 6 µg of LPS per mouse daily for 30 days and observed profound inhibitory effects on HSC activity and myeloid skewing [35]. We modified the method by reducing the amount of LPS to 1 µg and treated wild type and Id1 deficient mice. We show that this treatment dramatically but transiently stimulated the production of a number of inflammatory cytokines and significantly impaired the repopulating potential of wild type LT-HSC but had little impact on short-term repopulation. In contrast, ablation of the Id1 gene, whose expression can be induced by LPS in vivo, rendered the animals insensitive to the damage caused by LPS. Further examination of the properties of LT-HSC revealed that this treatment significantly increased the number of CD150^+^CD48^−^LSK cells while decreased the percentages of label-retaining cells and quiescent CD150^+^CD48^−^LSK cells in wild type but not Id1 deficient mice. Taken together, it appears that chronic TLR signaling impairs HSC maintenance by disturbing its quiescence. Id1 deficiency appears to cause resistance to LPS treatment, thus suggesting a potential involvement of Id1 in TLR-mediated HSC damage.

## Methods

### Mice

C57BL/6J (B6) and B6.SJL-Ptprc^a^Pepc^b^/BoyJ (B6-CD45.1) mice were originally obtained from Jackson Laboratories (Bar Harbor, ME) and propagated in house. Id1 deficient mice were generated by inserting EGFP upstream of the coding sequence in the Id1 gene and backcrossed for at least 10 generations [13]. Mice were used at 8–12 weeks of age. ROSA26-Stop-YFP mice were purchased from Jackson Laboratories and ROSA26-CreERT2 mice were obtained from Dr. Thomas Ludwig of Columbia University Medical School. Mice carrying both YFP and CreERT2 alleles were each treated with 5 mg of tamoxifen daily for 3 days before isolation of bone marrow cells.

Mice were housed in the Laboratory Animal Resource Center of the Oklahoma Medical Research Foundation in a specific pathogen free environment. This project was approved under the protocol S0165-1 by the Oklahoma Medical Research Foundation Institutional Animal Care and Use Committee. Minimal pain and suffering were involved in the experiments and mice were sacrificed by CO_2_ asphyxiation.

### LPS treatment of mice

Cohorts of age and gender-matched C57BL/6J and Id1 deficient mice were each intraperitoneally injected with 100 µl of lipopolysacchride (L2880, 055:B5, Sigma-Aldrich) at the concentration of 0.01 µg/µl in PBS for 30 days. Cohorts of age and gender-matched mice of each genotype were set aside as untreated controls. We chose to use these mice as negative controls to avoid a potential of introducing unknown inducers by repeated injection of the vehicle control. We believe that this is an appropriate control since the purpose of this experiment is to create an inflammatory condition and not to identify the inducer of inflammation. In addition, it has already been shown that injection of PBS for 30 days does not have any effects as those observed for LPS [35].

### Bone marrow analyses and cell sorting

Cell collection and staining were conducted in Hank's balanced salt solution supplemented with 2.5% fetal calf serum (H5F). Analysis of whole bone marrow (WBM) composition were conducted with phycoerythrin (PE) or allophycocyanin (APC)-conjugated antibodies against Mac-1 (M1/70), CD19 (1D3), B220 (RA3-6B2) and Gr-1 (1A8). Unless noted otherwise, antibodies were purchased from BD Biosciences (San Diego, CA). After antibody staining, dead cells were excluded by propidium iodide incorporation (Molecular Probes; Eugene, OR). FACS analyses were performed using LSRII with FACSDIVA and FlowJo softwares. MoFlo was used for cell sorting.

Lineage negative (Lin^−^) marrow was obtained by first labeling whole bone marrow with rat Ig against CD2 (clone RM2–5), CD3 (17A2), CD5 (53-7.3), CD8 (53-6.7), Mac-1, CD19, B220, Gr-1, NK1.1 and the TER-119 antigen (BioLegend, San Diego, CA). Labeled cells were then incubated with BioMag anti-Rat Ig conjugated magnetic particles (Qiagen; Valencia, CA). Typically, a 10-fold enrichment was achieved. These cells were then stained with PerCP-Cy5.5-conjugated antibodies against the same lineage-specific markers and negative fractions were gated for further analysis.

To analyze HSC fractions, antibodies against lineage-specific markers were used together with APC-Cy7-anti-c-kit, PE-Cy5-anti-Sca-1, PE-anti-CD150, PE-Cy7-anti-CD48 and/or APC-anti-Flt3.

### Label-retaining Assay and Ki67 staining

Label-retaining assay was performed essentially as described [36], [37], except that LPS administration was included at the last 30 days for cohorts of wild type and Id1^−/−^ mice. BrdU labeling assays were performed using the APC-BrdU Flow kit (BD Biosciences) according to the manufacturer's instruction. Ki67 staining was carried out essentially as BrdU staining except that Alexa-Fluor647-anti-human Ki67 antibody (mouse-reactive, BD Biosciences) was used and DAPI was included to stain DNA.

### Transplant Assays

For transplant experiments, B6-CD45.1 recipient mice were preconditioned with two doses of 5.5 Gy in a Mark I gamma irradiator (J.L.Shepard & Associates; Glendale, CA). Competitive transplantation was performed by co-injecting each host with 1000 tester LSK isolated from five B6 or Id1^−/−^ treated or untreated mice with 1000 YFP positive LSK from ROSA26-stop-YFP/ROSA26-CreERT2 mice in which YFP expression was induced with tamoxifen. Cells to be transplanted were suspended in 200 µl H5F and injected intravenously. Secondary transplant was carried out with 2×10^6^ whole bone marrow cells pooled from primary recipients. At appropriate time points, marrow or peripheral blood samples were evaluated as described for bone marrow analysis. Donor cells were identified by staining with APC-anti-CD45.2 (clone 104) and recipient cells were detected with PE-anti-CD45.1.

### Cytokine analysis

Peripheral blood was collected from C57BL/6J and Id1 deficient mice post LPS treatment at different time points and from untreated mice. Cytokine levels in serum samples were measured by using a pro-inflammatory cytokine multiplex assay kit from Meso Scale Discovery (Gaithersburg, MD) by the Proteomics facility at the Oklahoma Medical Research Foundation.

### Quantitative RT-PCR

Total RNA was isolated from cells using Trizol reagent and cDNA synthesis was carried out using MMLV reverse transcriptase and random primers (Life Technology, Grand Island, NY). Real-time PCR was performed using a Power SYBR Green PCR master mix on AB7500 system (Life Technology). Levels of expression were normalized against that of β-actin. Primers sets used were: Id1, CACCCTGAACGGCGAGAT and TTTTTCCTCTTGCCTCCTGAAG; Id2, ATGAAAGCCTTCAGTCCGGTG and AGCAGACTCATCGGGTCGT; SCL, CACTAGGCAGTGGGTTCTTTG and GGTGTGAGGACCATCAGAAATCT; Lyl1, CCCTCACCCCTTCCTCAACA and AGCCAAGTCCAGCTCACTATG. Primers used to detect TNFα were purchased from Qiagen (Valencia CA).

### Statistical analysis

Two-tailed Student's *t* test was used to determine the statistical significance.

## Results

### Chronic TLR signaling stimulates transient production of inflammatory cytokines

**Figure 1:**
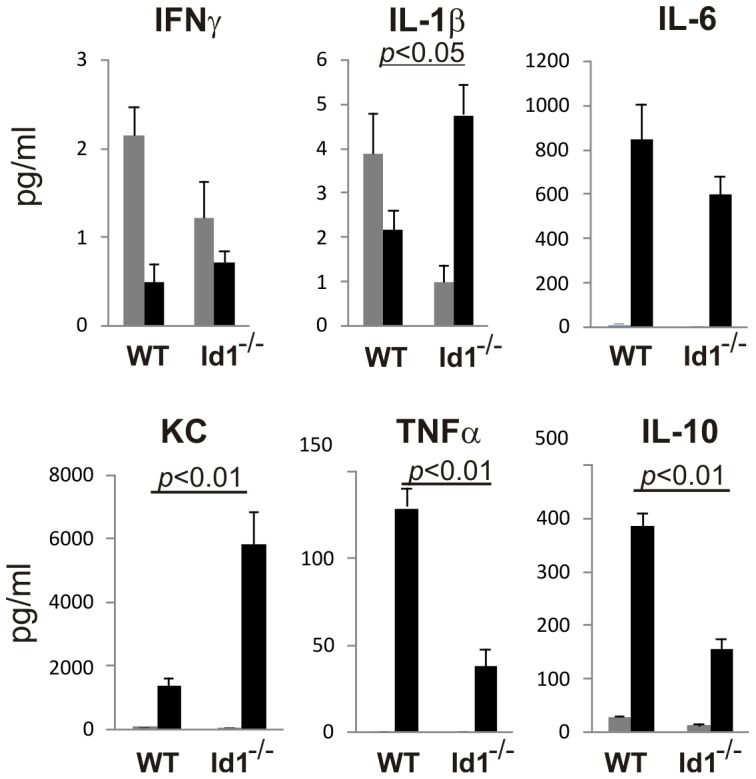
Cytokine production induced by chronic LPS stimulation. Serum samples (n>6) were collected from indicated strains of mice untreated (grey bar) or treated with a daily dose of 1 µg LPS for 15 days (black bar). Indicated cytokines were measured using a multiplex assay kit as described in Method. Data shown are averages with SEM. The levels of cytokines induced by LPS in WT and Id1^−/−^ mice were compared and statistically significant differences are indicated by showing the *p* values.

Signaling through toll-like receptors triggered by LPS is known to induce production of inflammatory cytokines by a variety of cell types in the body. To determine if chronic low dose LPS administration could elicit such cytokine responses, we measured the levels of a panel of inflammatory cytokines in the serum of wild type and Id1 deficient mice after a daily injection of 1 µg LPS per mouse over a course of 30 days. A pilot experiment was initially conducted by sampling serum taken on days 0, 15, and 30 of treatment. We found that a number of cytokines were dramatically stimulated on day 15, but the levels all returned to normal by day 30 (data not shown). Therefore, we focused on the comparison of cytokine levels in larger cohorts of animals treated for 15 days. As shown in Figure 1, while the oscillation of IFNγ and IL-1β levels was within several fold with or without LPS treatment, dramatic induction of IL-10, KC, TNFα and IL-6 was detected following LPS injection in either wild type or Id1 deficient mice. However, wild type mice produced significantly higher levels TNFα and IL-10 but lower levels of KC and IL-1β compared to Id1 deficient mice, suggesting an intrinsic difference between the systemic responses to LPS in wild type and Id1 deficient mice. Moreover, these results indicate that the prolonged low dose LPS treatment indeed elicits inflammatory responses in the animals.

### LPS stimulates Id1 expression in vivo

**Figure 2:**
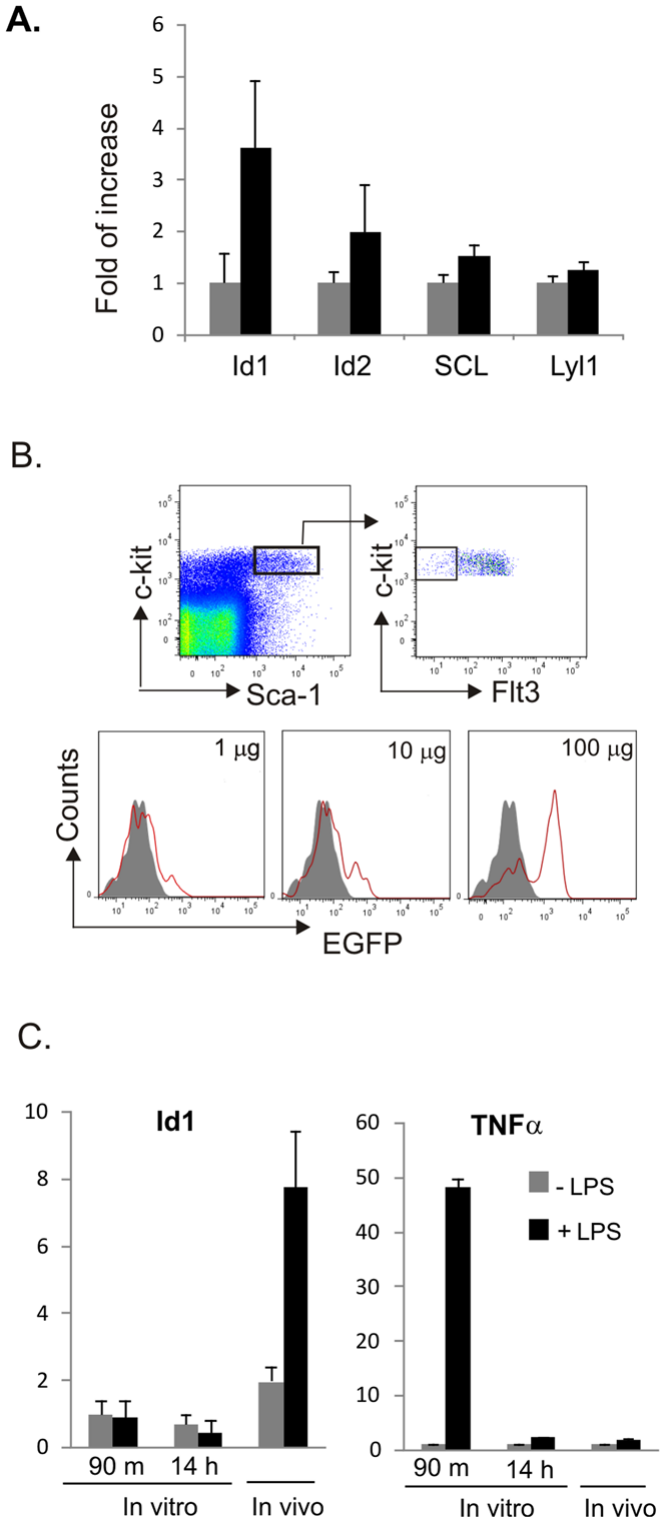
Induction of Id1 expression by LPS. (A) Induction of Id1 expression by LPS in vivo. Real-time RT-PCR analyses were performed on total RNA prepared from sorted Lin^−^c-kit^+^Flt-3^−^ bone marrow cells of wild type mice treated with (black bar) or without (grey bar) a single dose of 100 µg LPS for 48 hours. Relative levels of expression were shown as average fold of increase between untreated and treated samples with SD. (B) Expression of Id1 as indicated by EGFP fluorescence in Id1 deficient mice. EGFP expression is driven by the Id1 promoter in the knock-in construct. The Flt-3^−^LSK population was gated as diagrammed on the top. EGFP fluorescence of the gated cells was compared between untreated (shaded) and treated (red line) with indicated doses of LPS for 48 hours. (C) Induction of Id1 expression in vitro. LSK cells were sorted from wild type mice and incubated in medium containing SCF and Flt3-L for 6 hours. LPS was then added at a concentration of 1 µg/ml and incubated for indicated length of time. No treatment controls were set up for each time point. Samples from in vivo treatment are as described for (A). Relative levels of expression of indicated transcripts were shown as average fold of increase between untreated and treated samples with SD.

We have previously shown that Id1 expression in HSC/multipotent progenitors (LSK) can be stimulated by cytokines such as IL-3, IL-6 and GM-CSF in vitro. [16] To test if LPS treatment has similar effects, we first injected wild type mice with one dose of 100 µg of LPS and isolated bone marrow cells 48 hours later. Because the Sca-1 marker is known to be up-regulated under acute inflammation, we used an alternative strategy, the Lin^−^c-kit^+^Flt3^−^ population, to enrich for HSC and multipotent progenitors. Total RNA was thus isolated from these cells for analysis of genes encoding possible inhibitors of E proteins (Figure 2A). Id1 was found to be more dramatically up-regulated by LPS than Id2, SCL and Lyl1. The levels of Id3 and Id4 were below the threshold of detection.

To further analyze Id1 expression, we made use of the EGFP gene knocked into the Id1 locus in Id1^−/−^ mice to examine Id1 expression 48 hours after a single dose of 1, 10 or 100 µg of LPS injection. As shown in Figure 2B, in LSK^−^ population was further fractionated based on Flt3 expression. We compared EGFP fluorescence in Flt3^−^LSK cells from mice treated with or without different doses of LPS. EGFP fluorescence in Id1^−/−^ mice increased proportionally to the amounts of LPS injected, thus suggesting that LPS treatment stimulates transcription of the Id1 gene. However, incubation of sorted LSK cells with LPS in culture for 90 minutes or overnight did not elevate Id1 expression (Figure 2C). As a positive control, TNFα expression was found to be dramatically stimulated by treatment with LPS for 90 minutes, demonstrating the ability of LSK cells to respond to LPS (Figure 2C). These results suggest the stimulatory effect of LPS on Id1 transcription in vivo is likely to be indirect.

### Chronic LPS treatment increases the number of phenotypic LT-HSC

**Figure 3:**
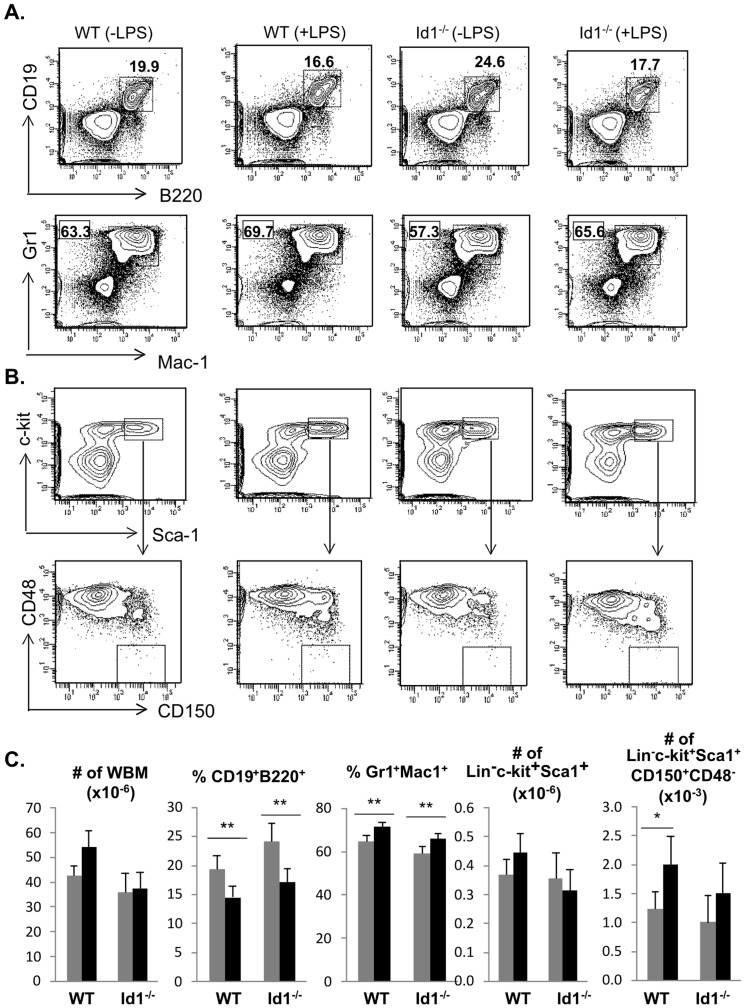
Examination of hematopoiesis after chronic LPS treatment. (A) Lymphoid and myeloid differentiation. Whole bone marrow cells from WT and Id1^−/−^ mice each treated with or without a daily dose of 1 µg of LPS for 30 days were stained with antibodies against indicated markers. Each cohort contains at least five animals. Lymphoid and myeloid cells were characterized as B220^+^CD19^+^ and Mac-1^+^Gr-1^+^, respectively. The percentages are shown next to the gates. (B) Analysis of phenotypic HSC. Lineage negative bone marrow was stained with antibodies against c-kit, Sca-1, CD150 and CD48. The LSK fraction shown in the top row was further analyzed for expression of CD150 and CD48, and the CD150^+^CD48^−^ subset was considered phenotypic HSC. (C) Average numbers or percentages of indicated subset of bone marrow are shown with SD. ** p<0.01; *p<0.05.

To examine the effects of prolonged low dose injection of LPS on hematopoiesis, we analyzed the bone marrow of wild type and Id1 deficient mice treated with or without a daily dose of 1 µg of LPS for 30 days. We stained whole bone marrow cells with antibodies against B220 and CD19 for B lymphoid cells as well as Mac-1 and Gr1 for myeloid cells (Figure 3A). Consistent with reports in the literature [35], LPS administration led to a decrease in the proportion of lymphoid cells and increase in that of myeloid cells in both wild type and Id1 deficient mice even though the overall bone marrow counts did not change significantly (Figure 3C).

Furthermore, the lineage negative population were analyzed with antibodies against c-kit, Sca-1, CD150 and CD48 (Figure 3B). The Lin^−^c-kit^+^Sca-1^+^ (LSK) population contains HSC and multipotent progenitors and its numbers were not affected by LPS (Figure 3C). LPS treatment also did not alter the colony forming potential as well as the balance of myeloid versus lymphoid differentiation of LSK in vitro (data not shown). However, the number of CD150^+^CD48^−^LSK cells, in which long-term HSCs were thought to constitute 50% was significantly increased after LPS treatment of wild type but not in Id1 deficient mice, suggesting that LPS stimulated an expansion of wild type HSC (Figure 3C).

### Chronic LPS treatment impairs the long-term repopulating potential of wild type but not Id1 deficient HSC

**Figure 4:**
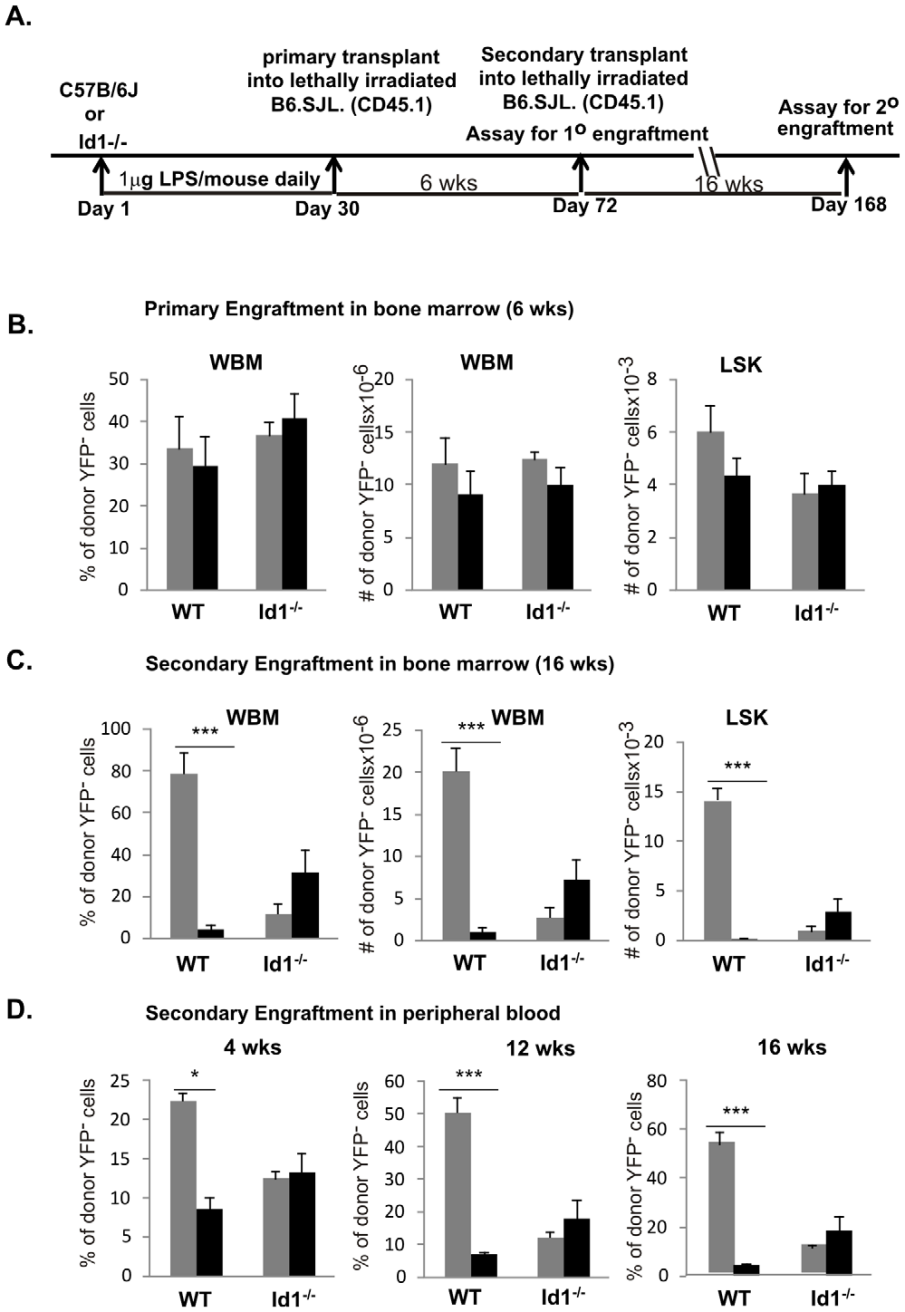
Competitive repopulation assay of HSC activity. (A) Experimental scheme. Lethally irradiated B6.CD45.1 recipients were transplanted with 1000 tester LSK from WT or Id1^−/−^ mice untreated and treated with a daily dose of 1 µg LPS for 30 days together with 1000 competitor LSK from ROSA26-stop-YFP/ROSA26-CreERT2 mice exposed to tamoxifen as described in the methods. Actions taken during the course of 168 days are indicated. (B) Analysis of primary engraftment in the bone marrow of recipients of LPS treated (black bar) and untreated (grey bar) donor LSK cells. Six weeks post transplant, mice were sacrificed and whole bone marrow (WBM) cells were collected. Donor engraftment in WBM and LSK populations were analyzed by staining with antibodies against CD45.2 donor-specific marker and determination of YFP fluorescence in CD45.2^+^ cells. Average percentages or numbers of donor-derived YFP negative cells are shown with SEM. (C) Analysis of secondary engraftment in the bone marrow. Pooled bone marrow from each cohort of primary recipients was transplanted into lethally irradiated recipient. Secondary recipients were sacrificed 16 weeks later. Bone marrow cells were interrogated as described for (B). (D) Analysis of peripheral blood from secondary recipients. Peripheral blood was taken 4, 12, and 16 weeks post secondary transplant. Engraftment of donor-derived YFP^−^ cells is shown as average percentage with SEM. *** p<0.001; *p<0.05.

We next examined the effects of the prolonged LPS exposure on HSC function in wild type and Id1 deficient mice. We harvested bone marrow from cohorts (n>6) of wild type or Id1^−/−^ mice with or without a 30-day treatment of LPS. LSK stem/progenitor cells were isolated from pooled bone marrow cells within each cohort and used as testers for transplantation assays (Figure 4A). The YFP^+^ LSK cells were used as competitors and were isolated from ROSA26-stop-YFP/ROSA26-CreERT2 mice, which were induced with tamoxifen for YFP expression. We intravenously injected lethally irradiated CD45.1^+^ recipients with 1000 LSK testers along with 1000 YFP^+^LSK competitors. This experimental design allows unambiguous identification of tester, competitor and host-derived cells. Six weeks later, cohorts of primary transplant recipients were sacrificed and bone marrow cells were collected to measure short-term reconstitution, an experimental scheme well-established by Eaves and colleagues [38]. Bone marrow cells were stained with antibodies against CD45.2 along with those for lineage specific markers. Primary engraftment was analyzed by first gating on donor-derived CD45.2^+^ bone marrow cells, which constituted over 60% of the whole bone marrow cells at this time point. These cells were then examined for YFP expression. The percentage and number of YFP^−^ tester cells in total bone marrow were scored in Figure 4B. An equivalent fraction of YFP^+^ cells were detected (data not shown). No significant differences in the short-term reconstitution potential were found between treated and untreated animals and between wild type and Id1 deficient mice. Likewise, the engraftment of the LSK compartment was also similar in the primary recipients (Figure 4B). Moreover, no skewing towards myeloid differentiation was observed after LPS treatment (data not shown).

Next, whole bone marrow cells within each cohort of primary recipients were pooled and injected into lethally irradiated secondary recipients at a dose of 2×10^6^ cells per mouse. Secondary recipients were sacrificed 16 weeks later. In addition, peripheral blood was collected 4, 12 and 16 weeks after secondary transplant. Robust engraftment was observed in secondary recipients. CD45.2^+^ donor derived cells were then gated for YFP^−^ and YFP^+^ cells, which separate cells that originated from testers and competitors, respectively. Figure 4C shows the engraftment of YFP^−^ testers in the bone marrow 16 weeks post transplant. Untreated wild type donor cells reconstituted the hosts efficiently both in the percentage and number of whole bone marrow cells. However, LPS treatment greatly diminished the repopulating potential of long-term HSC. To rule out any possibility that the disparity is due to defects in lymphopoiesis or myelopoiesis, we determined the number of LSK multipotent progenitors and found a similar impairment (Figure 4C). This result is consistent with observations made in peripheral blood during the course of 16 weeks post secondary transplant (Figure 4D).

In contrast, although Id1 deficiency is known to cause a defect in LT-HSC function [13], LPS treatment did not further impair the long-term repopulating potential as determined by the levels found in whole bone marrow cells, LSK, and peripheral blood (Figure 4C and D). In fact, LPS treatment consistently resulted in a small increase in LT-HSC activity even though the difference was deemed statistically insignificant. Nevertheless, these results suggest that lack of Id1 renders the animal insensitive to LPS-mediated insult on HSC.

### Chronic LPS treatment decreases the number of label-retaining cells in wild type but not Id1 deficient mice

**Figure 5:**
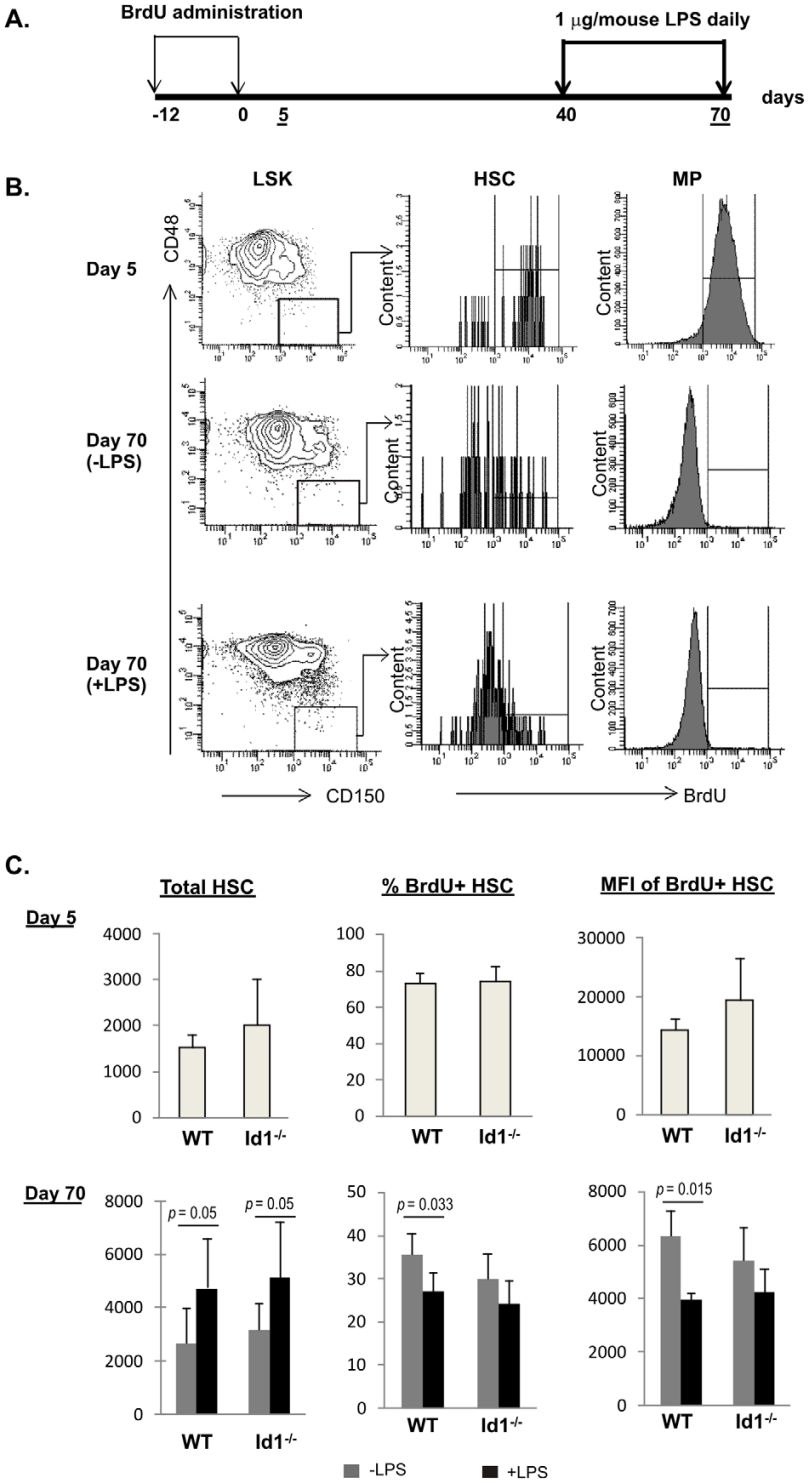
Label-retaining assay. (A) Experimental scheme. Time points (days) when actions were taken are labeled and those when mice were assayed are underlined. (B) FACS analysis of BrdU incorporation on Day 5 and Day 70. FACS plots of untreated and treated WT mice are shown as examples. BrdU incorporation in CD150^+^CD48^−^LSK (HSC) and Lin^−^c-kit^+^Sca-1^−^ myeloid progenitors (MP) populations are shown in histograms. BrdU positive gates were set with the same subsets of cells from control mice not exposed to BrdU. (C) Average values of HSC count, percent of BrdU^+^ HSC, and the mean fluorescence intensity (MFI) of BrdU^+^ cells in each cohort of 5 mice at indicated time point are shown with SD. Grey bar, untreated; black bar, treated daily with 1 µg of LPS for 30 days.

To understand the mechanism by which LPS impairs HSC activity, we examined the proliferative states after LPS treatment by using a label-retaining assay developed by Trumpp and colleagues as diagrammed in Figure 5A [36], [37]. Wild type and Id1 deficient mice were labeled with BrdU by two daily doses of intraperitoneal injection of BrdU followed by providing BrdU-containing drinking water for 10 days (Figure 5A). Five days later, cohorts were sacrificed to determine the efficiency of BrdU incorporation while the remaining cohorts were maintained until day 40 post BrdU labeling. At this time point, two cohorts of each genotype were set up to be treated with or without a daily dose of LPS for 30 days. To determine BrdU labeling in HSC on Days 5 and 70, we stained lineage-negative bone marrow cells with antibodies against c-kit, Sca-1, CD150 and CD48 in addition to intracellular staining for BrdU. LSK cells were analyzed for CD150 and CD48 expression and CD150^+^CD48^−^ HSC cells were gated. Levels of BrdU in these cells were then analyzed in a histogram and the BrdU positive gate was set by using negative control cells from mice not exposed to BrdU. Figure 5B shows the analyses of wild type HSCs as examples. In addition, as a control for the specificity of BrdU staining, we analyzed BrdU incorporation by the Lin^−^c-kit^+^Sca-1^−^ MP population, which include subsets of myeloid progenitors. While the majority of MP cells were positive for BrdU on Day 5, they have lost BrdU by Day 70 due to their rapid proliferation. This result thus boosted the confidence of the analysis.

Figure 5C summarizes the results of BrdU labeling analysis of HSC with or without LPS exposure. Both wild type and Id1 deficient HSC were labeled efficiently five days after BrdU administration, displaying 73% and 74% BrdU positive cells, respectively. The mean fluorescence intensity (MFI) of the BrdU^+^ cells was in the range of 15,000 to 20,000. By Day 70, we observed an increase in total number of HSC after LPS treatment of both wild type and Id1 deficient mice. However, the percentage of HSC which retained BrdU label was significantly higher in untreated than treated wild type HSC. Furthermore, the MFI of the BrdU^+^ population was also higher in untreated cells. These findings are consistent with the notion that LPS treatment increased the turnover of HSC. In contrast to wild type HSC, LPS stimulation did not appear to significantly alter the percentage of label-retaining cells or the MFI of BrdU^+^ cells in Id1 deficient mice (Figure 5C). Slightly lower percentages and MFI were observed for both untreated and treated Id1 deficient HSC in comparison to wild type untreated cells, but the differences were statistically insignificant.

### Chronic LPS treatment decreases the quiescence of wild type but not Id1 deficient HSC

**Figure 6:**
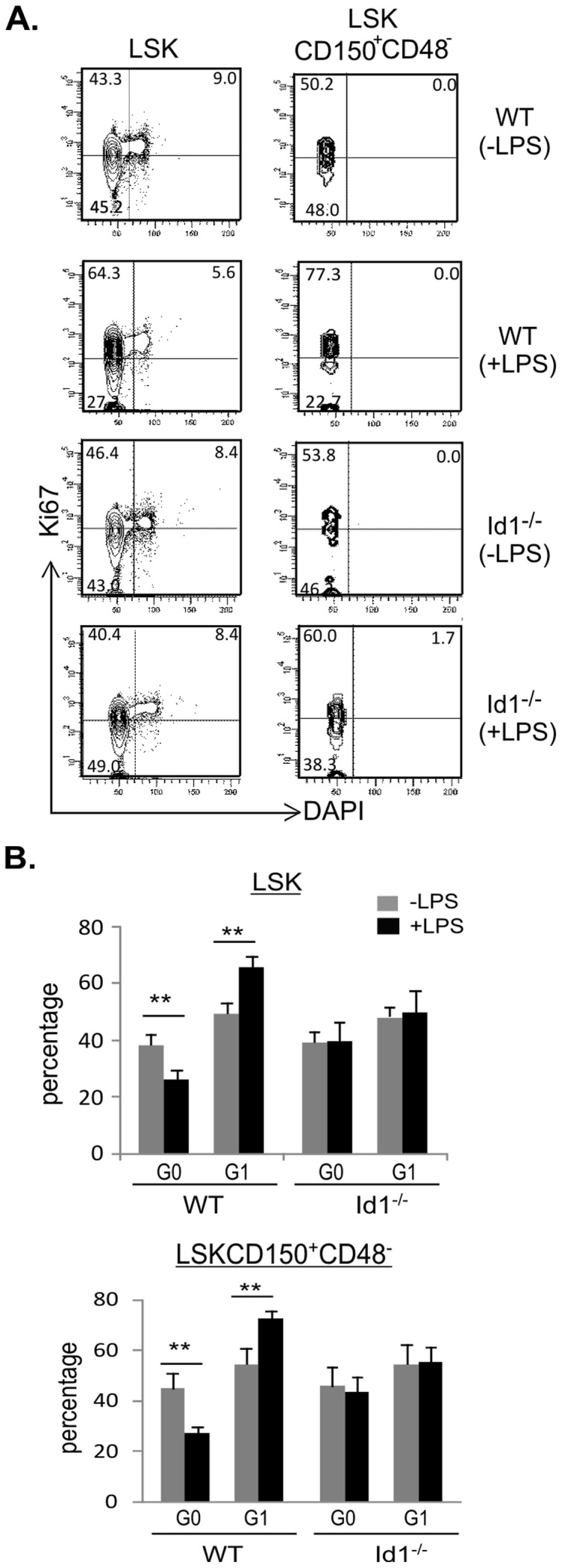
Analysis of cell cycle state of HSC. (A) FACS analysis for expression of Ki67 in indicated subsets were performed by intracellular staining followed by DAPI staining for DNA content. Ki67 positive gate for each sample was set based on the level of Ki67 in cycling cells identified according to DAPI intensity of cells in the LSK population. Numbers indicate the percentage in each quadrant. (B) Average percentages of cells in G0 and G1 phases are shown with SD. Grey bar, untreated; black bar, treated with 1 µg of LPS for 30 days. ** p<0.01.

Results from the label-retaining assay suggested that LPS treatment might have stimulated the proliferation of wild type HSC. If so, the quiescence of HSC could be disturbed and more cells will have exited the G0 state of the cell cycle. We, therefore, stained HSC for Ki67, whose expression inversely correlates with the G0 state. HSC populations were defined as described in Figure 5 and intracellular staining with anti-Ki67 was carried out followed by incubation with DAPI (Figure 6A). The LSK population was also analyzed as a control and the Ki67 positive gate was determined based on the intensity of Ki67 of cells in the S and G2/M phases. The same gate was used to analyze HSC populations. As summarized in Figure 6B, LPS treatment of wild type mice resulted in a decrease of the percentage of cells in G0 and a corresponding increase in that in G1 phase. These alterations occurred in both LSK and HSC populations. In contrast, LPS exposure did not have significant effects on these populations in Id1 deficient mice. Taken together, these results are consistent with the idea that LPS treatment disturbs the quiescence of HSC in wild type mice.

### Chronic LPS treatment does not enhance apoptosis of HSCs

**Figure 7:**
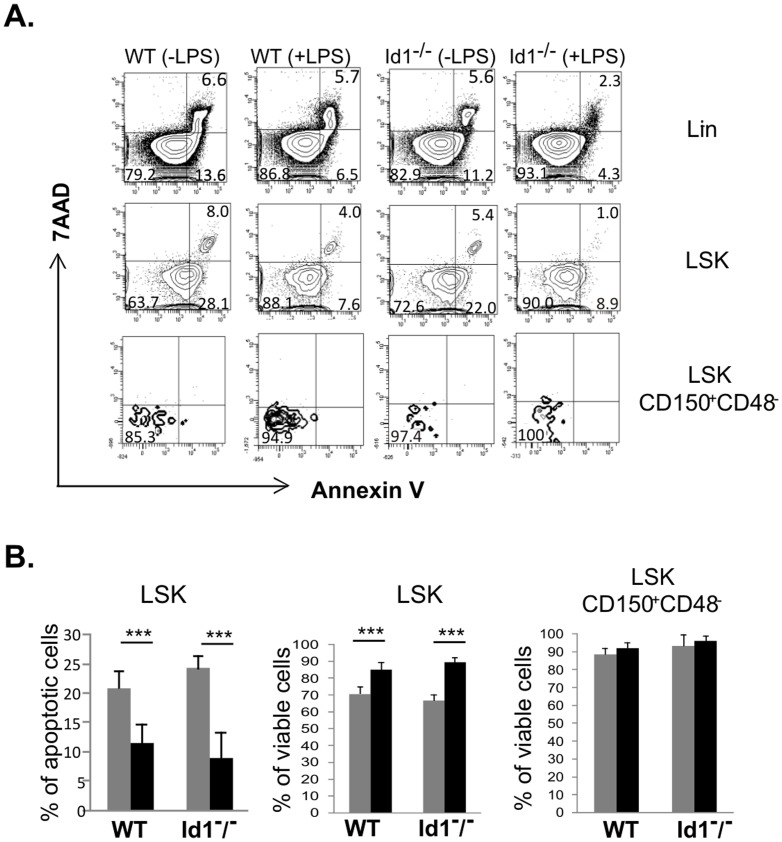
Analysis of apoptosis. (A) FACS analysis of indicated subsets was performed by Annexin-V and 7AAD staining. The Annexin-V positive gate was set based on its intensity on 7AAD^+^ Lin^−^ cells, which represent Annexin-V positive dead cells. Numbers indicate the percentage in each quadrant. (B) Average percentages of Annexin-V^+^7AAD^−^ or Annexin-V^−^7AAD^−^ (viable) cells are shown with SD. Grey bar, untreated; black bar, treated with 1 µg of LPS for 30 days. *** p<0.0001.

There is a formal possibility that increased HSC turnover of wild type HSCs detected using the label retaining assay is due to increased apoptosis induced by LPS. To address this issue, we stained cells with Annexin-V and 7AAD. Annexin-V^+^7AAD^−^ cells are considered apoptotic whereas Annexin-V^+^7AAD^+^ ones are dead cells. As shown in Figure 7A, while apoptotic cells are detectable in both Lin^−^ and LSK populations, very few apoptotic cells could be found in the HSC fraction. LPS treatment did not increase but decreased the percentages of apoptotic cells. Statistical analyses showed that the percentages of apoptotic LSK cells were significantly decreased after LPS treatment of both wild type and Id1 deficient mice (Figure 7B). Since very few apoptotic HSCs were detectable, we quantified the percentages of viable HSCs (Annexin-V^−^7AAD^−^) and found them to be similar in treated and untreated wild type or Id1^−/−^ mice (Figure 7B). It is not clear how LPS stimulation facilitated cell survival but it is important to note that LPS did not increase cell death.

## Discussion

LPS binds to TLR4 to elicit cellular inflammatory responses. By administering a low dose of LPS daily for 30 days, we intended to mimic inflammatory conditions similar to those occurring during chronic pathogenic infection or autoimmunity. Indeed, we detected dramatic induction of a number of inflammatory cytokines such as IL-6, KC, TNFα and IL-10 on Day 15 of the treatment, but cytokine levels returned to normal at the time when HSC were analyzed. It should be noted that the analysis on Day 15 serves as a snap shot of the inflammatory responses triggered by LPS. Expression of other cytokines tested may also be affected, but the induction follows a different kinetics. Furthermore, LPS may stimulate the production of additional cytokines which were not included in our assay. Whether this burst of inflammatory cytokines has any adverse effects on stem cells remains to be tested. However, several such cytokines are known to have direct effects on the properties of HSC. For example, IL-6 and IL-10 have stimulatory effects on HSC self-renewal whereas TNFα exerts inhibitory effects [39]–[42]. Apart from the roles of inflammatory cytokines, HSCs are also found to express TLR4 and, thus, have the potential to directly bind LPS, which initiates downstream signaling within HSC [43], [44]. Our current data cannot distinguish the HSC-intrinsic from cytokine-mediated effects on HSC maintenance after LPS treatment, although it is likely that both mechanisms are at play.

Regardless the mechanisms by which LPS exerts its deleterious effect on HSC activity, we have shown that chronic LPS treatment profoundly impairs the repopulating potential of wild type HSC. This defect is accompanied by a significant increase in the number of phenotypic HSC and a reduction in the percentage of label-retaining cells, which could be interpreted to mean that an increased turnover of LT-HSC occurred. Since the viability of HSC was unchanged when assayed after the 30-day LPS treatment, the increased turnover is mostly likely due to accelerated proliferation. Although BrdU labeling used in the label-retaining assay has been thought to disturb the quiescence of HSC [37], data from examination of Ki67 levels in HSC by intracellular staining also suggested that LPS treatment directs the cells out of the G0 state. LT-HSCs are normally present in a quiescent state residing in their niches in the bone marrow [45]. Loss of quiescence is believed to be detrimental to stem cell integrity and eventually leads to stem cell exhaustion [12]. Therefore, increased HSC cycling stimulated by LPS could compromise the ability of HSC to reconstitute the bone marrow of transplant recipients. At the dose of 1 µg of LPS per mouse, although we detected a slight myeloid skewing in treated animals, we did not find the same effect when treated HSCs were transplanted into primary or secondary recipients, which was observed when mice were treated with 6 µg of LPS [35]. Perhaps, the threshold is higher for upsetting the balance between lymphoid and myeloid potential of HSCs.

We also found that LPS treatment in vivo stimulated Id1 expression in Lin^−^Flt3^−^c-kit^+^ or Flt3^−^LSK cells, a population containing HSC as well as other progenitors. It is of note that incubation of LSK with LPS in vitro could not stimulate Id1 expression, which suggests that up-regulation of Id1 by LPS is likely mediated by inflammatory cytokines. More importantly, LPS injection into Id1 deficient mice did not appear to have any significant effects on HSC function and properties compared to control Id1 deficient mice, even though it led to skewing of lymphoid versus myeloid differentiation. This raises a possibility that the effects of LPS might be at least partially mediated by Id1 expression. Admittedly, Id1 deficiency itself markedly diminishes the long-term repopulating potential of HSC [13], [15]. We thought that loss of Id1 results in elevated E protein activities and pre-mature differentiation of LT-HSC into downstream lineages, thus exhausting the LT-HSC pool. Incidentally, one report did not reveal any HSC intrinsic defect in Id1 deficient mice using a less stringent measurement [29]. In the current study, we found that LPS administration not only failed to further reduce but also somewhat elevated the engraftment capacity of Id1 deficient HSC. This apparent rescuing effect by LPS could be due to the induction of other Id proteins as we have found Id2 expression could be modestly induce by LPS (Figure 2). The effects of Id1 deficiency under normal and inflammatory conditions underscore the importance of maintaining an appropriate level of Id proteins for the wellbeing of HSC.

There are several mechanisms that could act separately or coordinately to render Id1 deficient mice insensitive to LPS-mediated effects. First, ablation of Id1 blunted the cytokine storm triggered by LPS. For example, Id1 deficient mice produced significantly lower levels of TNFα and IL-10 than wild type mice on Day 15 of LPS injection (Figure 1). The cell types predominantly contributing to the differences in systemic cytokine production in wild type and Id1^−/−^ mice are currently unknown although dendritic cells and macrophages are known to express Id1. Likewise, whether the difference in the levels of these cytokines contributes to the distinct responses of LPS between wild type and Id1 deficient HSC remains to be verified. Second, the LPS insensitivity of Id1^−/−^ mice could be HSC intrinsic. Id proteins are inhibitors of E proteins which play an important part in maintaining stem cell quiescence [17], [19]. Disruption of the E protein genes, E47, led to a hyperproliferative phenotype of HSC and decreased long-term repopulating potential [23]. These are reminiscent of the situations after LPS treatment of wild type mice, in which Id1 may be up-regulated and E protein function is inhibited. Finally, Id proteins are also expressed in many of the cell types that make up the bone marrow niche, including osteoblasts, endothelial cells, mesenchymal stem cells and macrophages [16], [27], [46], [47]. LPS could alter the behavior of these cells by increasing Id1 levels and, thus, create unfavorable environments for HSC to live in. Taken together, the intimate relationships between Id1 expression and inflammation, as well as HSC and their niches, make Id1 one of the plausible mediators of the effects on HSCs during inflammation, which can be elicited by LPS or other pathogenic agents.

LT-HSCs can be subdivided into dormant and activated populations, and hematopoietic stresses such as bone marrow injury or mobilization with G-CSF can activate dormant HSCs to self-renew [36], [48], [49]. Undoubtedly, a balance between the numbers of dormant and activated HSCs is crucial for the maintenance of a healthy pool of stem cells [50]. Up-regulation of Id1 by inflammatory stimuli could lead to disruption of HSC quiescence and increases in proliferation. Repeated occurrence of such scenarios could deplete the dormant fraction of HSC, which would be detrimental for the longevity of HSC. Parenthetically, it remains to be determined if periodical stimulation of Id1 expression throughout life is beneficial for rejuvenating HSC. After all, Id1 is necessary for maintaining steady-state levels of LT-HSC.
